# Henle fiber layer hemorrhage associated with combined central retinal vein occlusion and cilioretinal artery occlusion: a case report

**DOI:** 10.1186/s13256-023-04100-y

**Published:** 2023-08-20

**Authors:** Hamid Riazi-Esfahani, Nazanin Ebrahimiadib, Nikoo Hamzeh, Kaveh Fadakar, Elias Khalili Pour

**Affiliations:** Retina Service, Farabi Eye Hospital, Tehran University of Medical Sciences, Qazvin Square, South Kargar Street, Tehran, Iran

**Keywords:** Henle fibre layer hemorrhage, Central retinal vein occlusion, Cilioretinal artery occlusion

## Abstract

**Background:**

The purpose of this study is to describe a patient who experienced simultaneous central retinal vein and cilioretinal artery occlusions, as well as perifoveal hemorrhage in the Henle fiber.

**Case presentation:**

A 67-year-old Iranian woman presented with a 3-day history of reduced vision in her left eye. Venous tortuosity and retinal hemorrhage were observed in the retina, together with whitened regions around the fovea, consistent with the diagnosis of central retinal vein occlusion in conjunction with cilioretinal artery occlusion. In structural and *en face* optical coherence tomography, star-shaped hemorrhages were observed around the fovea, which looked hyperreflective in the Henle fiber layer.

**Conclusions:**

We present a case of central retinal vein occlusion exacerbated by cilioretinal occlusion and hemorrhage in the Henle fiber layer. The hemorrhage is most likely the result of increased intraluminal pressure in the deep capillary plexus.

## Background

Concurrent blockage of the central retinal vein and cilioretinal artery is uncommon but can result in severe visual loss. It has been postulated that blockage of the central retinal vein (CRVO) results in a significant rise in intraluminal pressure at the capillary level, which can result in the cilioretinal artery occlusion (CILRAO). In comparison to the central retinal artery, the cilioretinal artery is devoid of autoregulatory systems, making it susceptible to functional obstruction [1].

The purpose of this report is to describe a case with simultaneous CRVO and CILRAO associated with outer hemorrhagic Henle maculopathy, which we hypothesize occurred as a result of elevated retinal capillary pressure.

## Case presentation

**Fig. 1:**
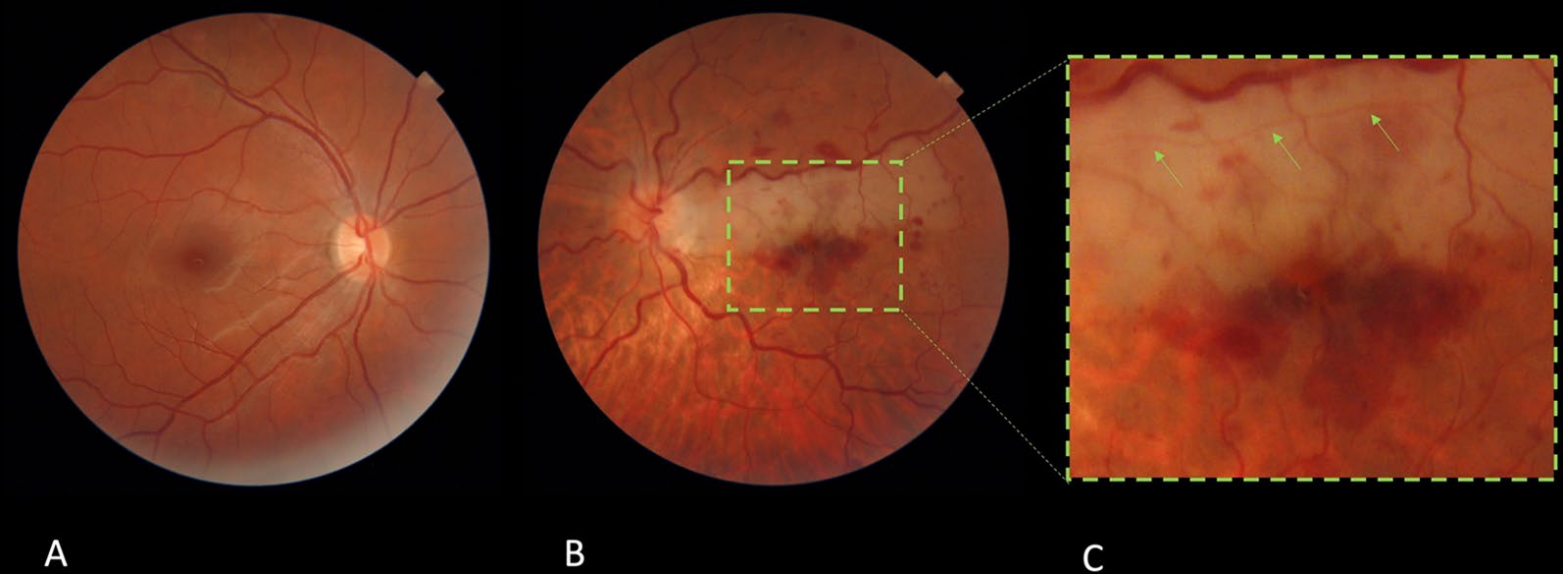
Fundus photograph of the right eye (**A**). Fundus photography was performed 3 days following the start of diminished visual acuity in the left eye. Engorged and tortuous retinal veins, as well as intraretinal bleeding, are diagnostic of CRVO (**B**). The region of whitening in the cilioretinal artery zone is consistent with a secondary diagnosis of cilioretinal artery blockage. The cilioretinal artery’s course is indicated by green arrows. A radial hemorrhage surrounding the fovea is seen (**C**)

A 67-year-old Iranian woman was sent to the retina clinic 3 days ago with a complaint of decreased vision of counting fingers in her left eye. Her prior medical history revealed no evidence of hypertension, diabetes, or cardiovascular disease, as well as no history of drug consumption. The spherical equivalent was −0.5D in both eyes (OU). Intraocular pressure was 14 mmHg OU. The fundus examination revealed engorged and tortuous retinal veins, as well as intraretinal hemorrhage, both of which were consistent with CRVO. There was a whitening in the cilioretinal artery zone, consistent with the diagnosis of cilioretinal artery occlusion. Additionally, hemorrhage encircled the fovea inferiorly in a radial pattern (Fig. 1).

Spectral-domain optical coherence tomography (SD-OCT) revealed a minor increase in retinal thickness. Additionally, a hyperreflective band was observed in the area of CILRAO, including both the superficial and middle retinal layers, but the lesion’s lower border looked hyperreflective only in the middle retinal layer, consistent with the diagnosis of paracentral acute middle maculopathy (PAMM). In the Henle layer, an oval-shaped hyperreflective region around the foveal center was found, corresponding with the radially oriented bleeding observed in fundus imaging.

*En face* optical coherence tomography (OCT) segmentation at the level of the deep capillary plexus (DCP) revealed a hyperreflective region in the distribution of the cilioretinal artery as well as base-out wedge-shaped hyperreflective lesions pointing to the fovea, which corresponded to the oval-shaped hemorrhages. In the area exclusively affected by venous occlusion, fern-like perivenular hyperreflective lesions were also observed inferotemporal to the fovea.

**Fig. 2:**
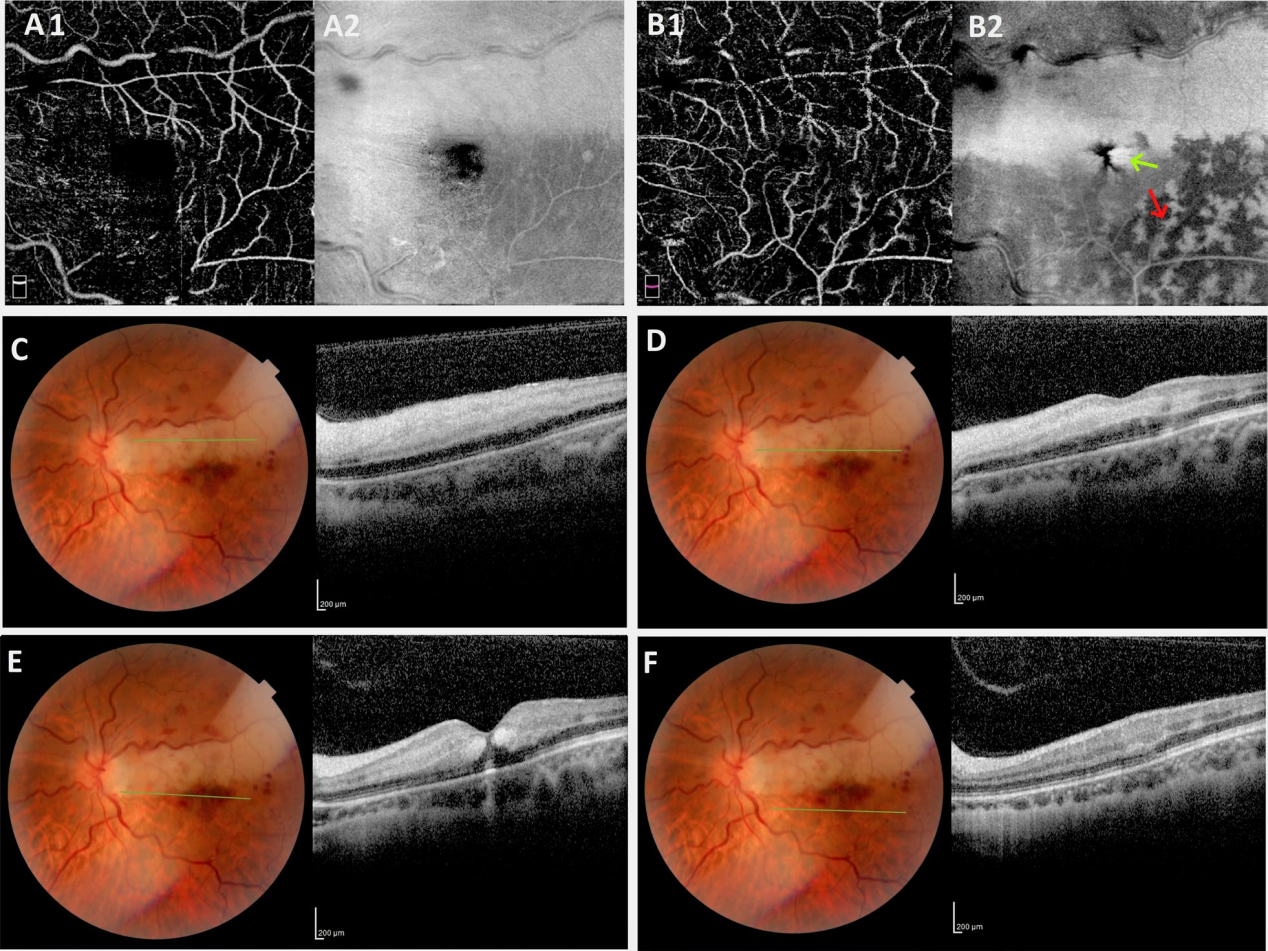
A slight reduction in vascular density in the superficial capillary plexus (SCP) and DCP can be seen on optical coherence tomography angiography (OCTA) of the affected eye (**A1**, **B1**). En face OCT segmented at the SCP level demonstrates mild hyperreflectivity around the area corresponding to the distribution of cilioretinal artery occlusion due to globular PAMM (**A2**). The DCP slab exhibits the projection artifact of superficial vessels (**B1**). *En face* OCT segmented at the DCP level demonstrates a diffuse hyperreflective area corresponding to the distribution of cilioretinal artery occlusion due to globular PAMM (**B2**), as well as small wedge-shaped hyperreflective areas surrounding the fovea corresponding to radial hemorrhages in the Henle fiber layer (green arrow). Additionally, fern-like perivenular hyperreflective lesions are seen in the inferior portion of the fovea in the region affected only by venous occlusion, consistent with fern-like PAMM (red arrow). B-scan SD-OCT demonstrates a slight increase in retinal thickness, as well as a diffuse hyperreflective band above the fovea in the area of CILRAO, which involves both the superficial and middle retinal layers (**C**), whereas the lesion’s lower border appears hyperreflective only in the middle retinal layer, consistent with PAMM (**D**). In the Henle fiber layer, a teardrop-shaped hyperreflectivity heading toward the foveal center is visible, matching to the radially oriented hemorrhage in the fundus photograph (**E**). SD-OCT B-scan from the inferior fovea demonstrates a dispersed hyperreflective band in the middle retinal layer, which corresponds to the region with fern-like perivenular PAMM in *en face* OCT (**F**)

*En face* OCT segmented at the DCP level demonstrates a diffuse hyperreflective area corresponding to the distribution of cilioretinal artery occlusion due to globular PAMM (Figs. 2B2, 3).

**Fig. 3:**
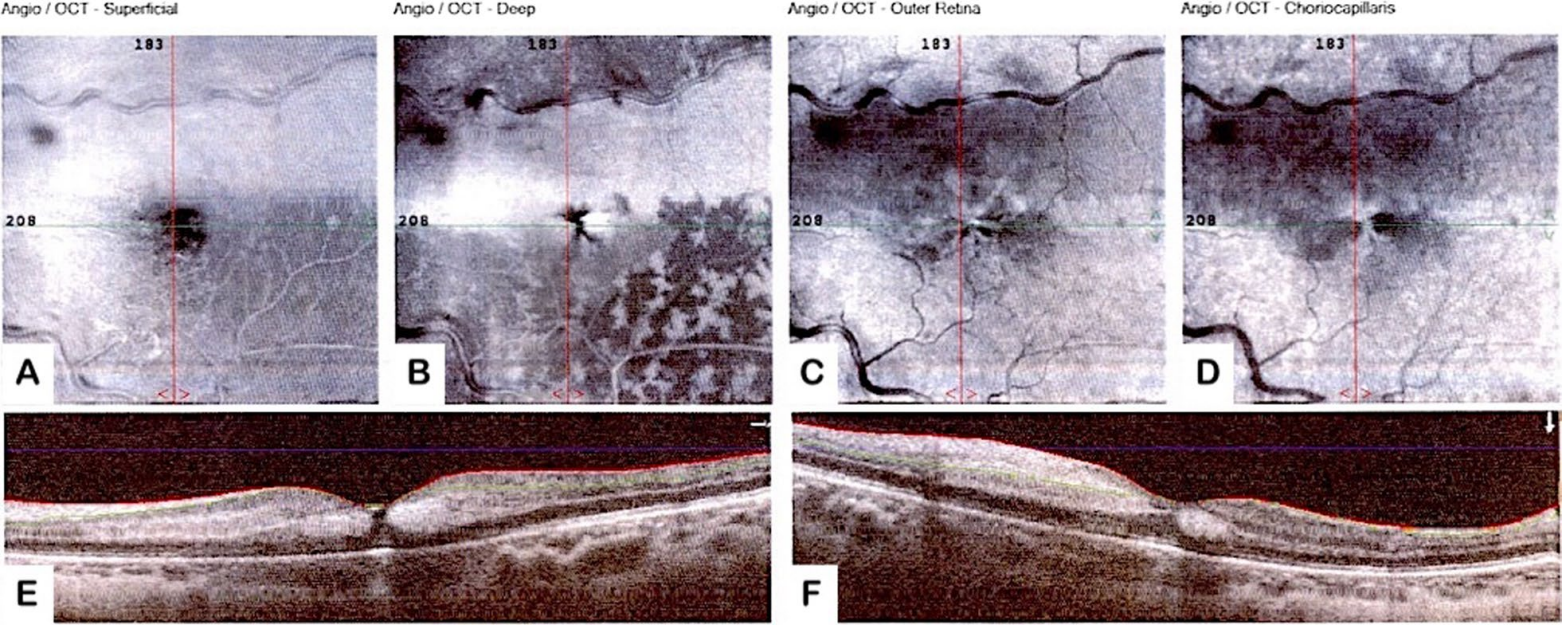
Displays *en face* optical coherence tomography (OCT) images of the left eye, specifically at the superficial capillary plexus level (**A**), deep capillary plexus level (**B**), outer retina level (**C**), and choriocapillaris level (**D**). The foveal region is imaged using horizontal (**E**) and vertical (**F**) optical coherence tomography (OCT) slabs. A vertical slab that travels from the superior fovea to the inferior parts of the fovea reveals a superior hyperreflective region that corresponds to the distribution of cilioretinal artery occlusion caused by globular paracentral acute middle maculopathy (PAMM), which is not apparent in the inferior fovea

The right eye has 20/20 vision and normal fundus examination. Cardiology and laboratory evaluations revealed no abnormalities in the current patient.

## Discussion and conclusions

CILRAO occurs in 5% of non-ischemic CRVO patients as a result of physiologic blockage of the cilioretinal artery induced by a rapid fast rise in intraluminal pressure in the retinal capillary bed [1–3]. However, our case is unique due to the concurrent appearance of significant radial hemorrhage in the Henle layer. The pathophysiology of this condition may be traced back to the unusual arrangement of the retina’s capillary plexus. Advances in retinal imaging have increased our knowledge of the complex retinal vasculature and resulted in the identification of three levels of separate capillary plexuses: the superficial capillary plexus (SCP), the intermediate capillary plexus (ICP), and the deep capillary plexus (DCP). Numerous investigations employing optical coherence tomography angiography (OCTA) have established that the capillaries in DCP are organized radially and merge toward a central vortex-like vein that empties directly into a large venule [4, 5]. Garrity and coworkers hypothesized that, because of the anastomoses between SCP, ICP, and DCP, SCP and ICP may not behave as capillary units with independent afferent arterioles and efferent venules. Rather than that, draining of the whole retinal capillary system may occur mostly at DCP [6].

In our case, a rapid increase in venous intraluminal pressure, as shown by concurrent CILRAO, may have elevated the pressure over the acceptable range for the venous drainage system at DCP. This has resulted in distinctive radial hemorrhage in the Henle layer, which is most likely caused by DCP located on the outside part of the inner nuclear layer adjoining the outer plexiform layer. The Henle fiber layer in the macula is oblique, regular, and radially symmetric, which results in star or petaloid hemorrhage in this layer [7].

Simultaneous fern-like perivenular ischemia exhibited on the inferior region of *en face* OCT in our patient implies that ischemia is in its early stages. This observation emphasizes the temporary nature of central retinal vein blockage, since more persistent restriction of outflow would have resulted in the globular or diffuse pattern of ischemia shown on *en face* OCT images [8].

Baumal and colleagues have documented Henle hemorrhage in 33 eyes of 23 patients, 7 of whom had retinal vein occlusion (RVO) [9]. They linked this phenomena to DCP’s aberrant venous pressure and hypothesized that a persistent increase in venous pressure results in inner retinal hemorrhage caused by SCP. They reasoned that this discovery is consistent with the ischemic cascade observed in DCP, which begins with perivenular fern-like ischemia and progresses to globular and diffuse patterns with involvement of the inner retina and greater ischemia severity [10]. Additionally, the presence of both retinal nerve fiber layer and perivascular hemorrhage is related with decreased visual acuity compared with those with just deep hemorrhage [11]. The occurrence of Henle hemorrhage, the absence of widespread inner retinal hemorrhage, and the presence of the perivenular PAMM all support this concept, which implicates DCP as the initiator of hemorrhage and ischemia in our case.

The etiology of ischemia in older individuals is commonly attributed to atherosclerosis and emboli. The assessment of carotid artery stenosis necessitates the utilization of carotid ultrasound. Based on the evaluation conducted by the cardiology service, there were no findings of cardiac or carotid artery abnormalities in the present patient.

We present a case of CRVO exacerbated by cilioretinal occlusion and hemorrhage in the Henle fiber layer. The hemorrhage is most likely the result of increased intraluminal pressure in the deep capillary plexus. Prospective investigations are needed to elucidate the precise pathophysiological process behind this pattern of bleeding and its effect on visual prognosis and treatment response.

## Data Availability

The datasets used in the current study are available upon reasonable request.

## Abbreviations

CRVO: Central retinal vein occlusion
CILRAO: Cilioretinal artery occlusion
OCT: Optical coherence tomography
SD-OCT: Spectral-domain optical coherence tomography
PAMM: Paracentral acute middle maculopathy
DCP: Deep capillary plexus
OCTA: Optical coherence tomography angiography
SCP: Superficial capillary plexus
ICP: Intermediate capillary plexus
